# Scoping review: national monitoring frameworks for social determinants of health and health equity

**DOI:** 10.3402/gha.v9.28831

**Published:** 2016-02-05

**Authors:** Leo Pedrana, Marina Pamponet, Ruth Walker, Federico Costa, Davide Rasella

**Affiliations:** 1 ISC Instituto de Saúde Coletiva, UFBA – Universidade Federal da Bahia, Salvador, Brazil; 2 Department of Evolution, Ecology and Behaviour, University of Liverpool, Liverpool, UK

**Keywords:** scoping review, social determinants, health inequity, equity, inequality, indicators, monitoring, framework, EQuAL

## Abstract

**Background:**

The strategic importance of monitoring social determinants of health (SDH) and health equity and inequity has been a central focus in global discussions around the 2011 Rio Political Declaration on SDH and the Millennium Development Goals. This study is part of the World Health Organization (WHO) equity-oriented analysis of linkages between health and other sectors (EQuAL) project, which aims to define a framework for monitoring SDH and health equity.

**Objectives:**

This review provides a global summary and analysis of the domains and indicators that have been used in recent studies covering the SDH. These studies are considered here within the context of indicators proposed by the WHO EQuAL project. The objectives are as follows: to describe the range of international and national studies and the types of indicators most frequently used; report how they are used in causal explanation of the SDH; and identify key priorities and challenges reported in current research for national monitoring of the SDH.

**Design:**

We conducted a scoping review of published SDH studies in the PubMed^®^ database to obtain evidence of socio-economic indicators. We evaluated, selected, and extracted data from national scale studies published from 2004 to 2014. The research included papers published in English, Italian, French, Portuguese, and Spanish.

**Results:**

The final sample consisted of 96 articles. SDH monitoring is well reported in the scientific literature independent of the economic level of the country and magnitude of deprivation in population groups. The research methods were mostly quantitative and many papers used multilevel and multivariable statistical analyses and indexes to measure health inequalities and SDH. In addition to the usual economic indicators, a high number of socio-economic indicators were used. The indicators covered a broad range of social dimensions, which were given consideration within and across different social groups. Many indicators included in the WHO EQuAL framework were not common in the studies in this review due to their intersectoral and interdisciplinary nature.

**Conclusions:**

Our review illustrates that the attention to SDH monitoring has grown in terms of its importance and complexity within the scientific health literature. We identified a need to make indicators more wide-ranging in order to include a broader range of social conditions. The WHO EQuAL framework can provide intersectoral and interdisciplinary means of building a more comprehensive standardised approach to monitoring the SDH and improving equity in health.

## Introduction

The relevance of identifying the social determinants of health (SDH) in order to improve health equity, especially for disadvantaged sections of society, has been widely discussed in recent years. Since the World Health Organization (WHO) Commission on Social Determinants of Health (CSDH) in 2005, the topic of the relationship between social determinants and health inequalities (1) has grown in importance in relation to global health and within research related to policy-making processes (2, 3).

The 2011 Rio Political Declaration on SDH (4) focused on a balance between sustainable development and better quality of life for all and reported on the importance of monitoring trends in health inequities and impacts of actions to tackle them. Reliable measures of SDH are critical to implementing evidence-based policies that are more inclusive of and more sensitive to the different needs of the population. The importance of national monitoring of SDH has been widely acknowledged in the Millennium Development Goal (MDG) process: defining priorities and strategies to support countries in data collection, analysis, and reporting (5).

**Fig. 1:**
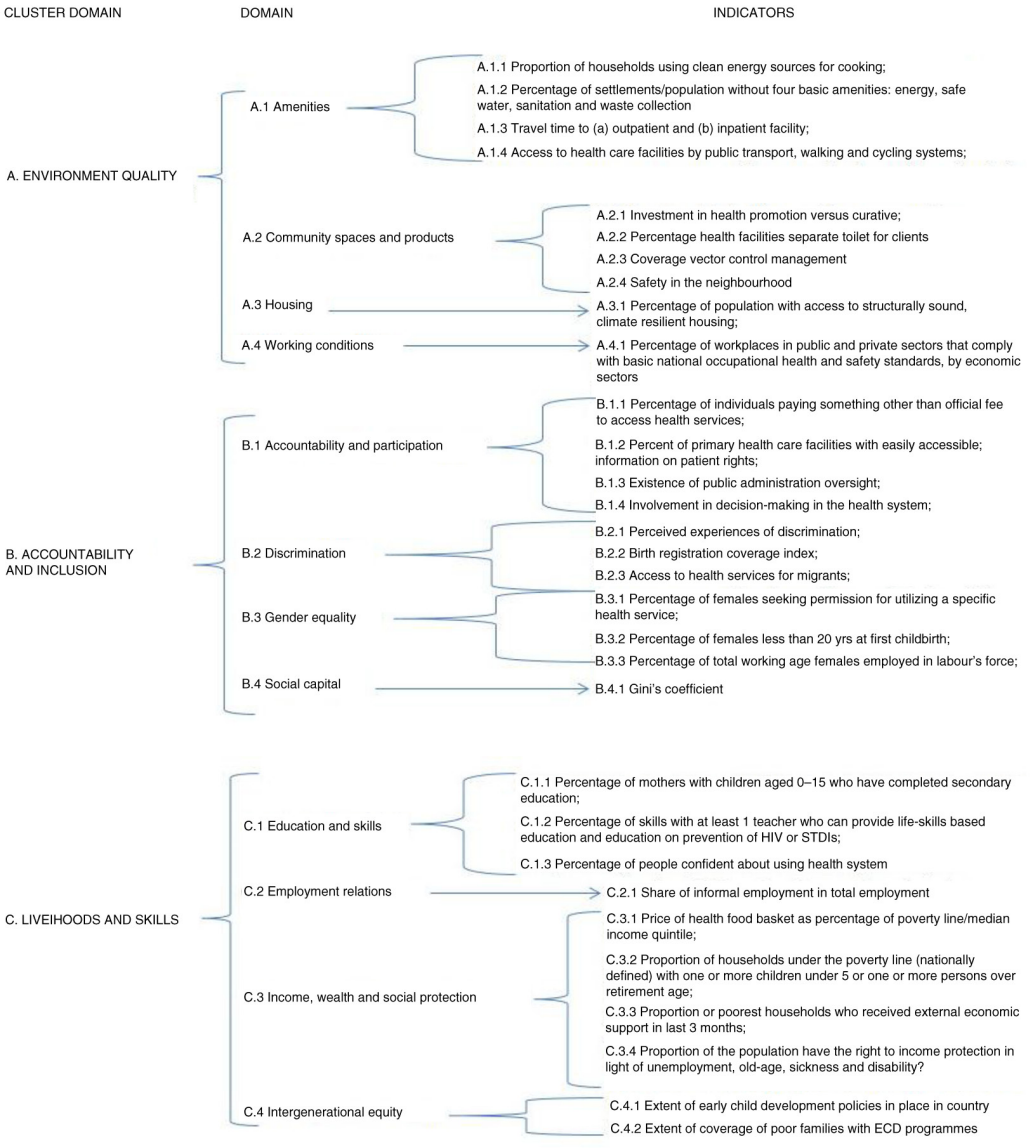
Cluster-domains, domains, and indicators of the EQuAL-WHO framework.

In 2013 the WHO, in collaboration with experts and researchers from several countries, began a project named *equity-oriented analysis of linkages between health and other sectors* (EQuAL). The main goal of this initiative was to identify possible approaches to complement the monitoring of equitable progress towards universal health coverage, focusing on intersectoral barriers and specific social determinants affecting health. The project used quantitative assessments, scoping and descriptive literature reviews, key informant opinion, and interviews and focus groups in case studies from four countries: Bangladesh, Brazil, South Africa, and Vietnam (6). One of the major outcomes of this initiative was the definition of a framework for 32 core and non-core indicators, which were classified into three cluster domains and 12 domains, using the EQuAL acronym: environment quality, accountability and inclusion, livelihoods and skills (Fig. 1) (unpublished).

In this article we present the results of a scoping review as an integrated part of the WHO EQuAL project and as a contribution to the main goal of identifying a set of indicators and approaches to use in monitoring the SDH and health equity at a national/country level. Because we did not know how the international scientific literature reported SDH indicators at a national/country level, we generated the following questions: How has the monitoring of SDH been presented at a national/country level in recent studies? Which indicators have been used? In which countries and for which populations and health diseases have these indicators been used? How have they been used for the analysis of SDH? Which dimension of the social determinants do they mostly represent?

The broad aim of this review was to construct a global representation of the domains and indicators used by recent research on SDH at a national level and analyse them in relation to the set of indicators proposed by the WHO EQuAL monitoring framework in early 2015.

The specific objectives were as follows:

- To describe the profile international studies in this area published in the last decade
- To describe the countries/nations that were the focus of these studies, their target populations, and their health problems
- To analyse and classify the indicators cited in the studies, specifically in relation to the EQuAL monitoring framework proposed by the WHO
- To describe indicators used to represent social determinants that have a larger negative impact on health
- To classify the priorities, challenges, and conclusions from current research on the national monitoring of SDH

## Methods

### Search strategy

A scoping review was used instead of a systematic review because of the complex exploratory nature of this study and the large volume of research that has used indicators and measurements of SDH (7). The assessment process was conducted by two reviewers. Both reviewers separately assessed the articles under the supervision of an expert in the field of systematic review and an expert in the SDH. For this study, we used PubMed as a source database. The database management software EndNote^®^ allowed us to store the citations identified in the search, to keep track of them, and to detect duplicates. A detailed review protocol is available upon request.

The search strategy focused on connections between the concepts of equity, SDH, and indicator monitoring. We allowed for possible variants by considering a number of descriptors in the search query – *unjustness*, *discrimination*, *equality/inequality*, *disparity*, *equity/inequity*, *coverage*. These terms were integrated with all possible word variations of *indicator monitoring* synonyms, such as *evaluation*, *indicator*, *measurement*, *monitoring*, and *assessment*. The search strategy was restricted to articles published from December 2004 to December 2014. The review considered only national scale studies. Reports were selected on a national, country, or regional territorial basis. Articles written in English, Italian, French, Portuguese, and Spanish were included. The final Medline search terms were as follows:

(unjustness OR discrimination OR inequalit* OR disparit* OR equit* OR inequit* OR equalit* OR coverage OR (social AND determinant*)) AND health [MeSH Terms] AND (national OR country OR regional) AND (evaluation* OR indicator* OR measurement* OR monitoring OR assessment) AND (“2004/12/07”[PDat]: “2014/12/04”[PDat]) AND (English[lang] OR Spanish[lang] OR Italian[lang] OR French[lang] OR Portuguese[lang])

### Screening process

We performed the first screen by reviewing titles and abstracts to select articles that met our inclusion criteria: national-level studies that mainly focused on monitoring social determinants and health by equity and that referenced at least one of the SDH indicators. Articles that did not present clear data in the abstract were included in the full text screen.

### Assessment and analysis criteria

After the first screen, a classification matrix was constructed based on the results of a pilot test conducted on a random sample of 10% of articles. The matrix was comprised of 13 categories corresponding to the specific objectives of our study. The studies were classified in relation to the language and year of publication, methodological approaches, statistical techniques, and visual presentations. Studies were also classified in relation to world region and country, the country income level (based on the World Bank's classification: www.data.worldbank.org/about/country-and-lending-groups#Upper_middle_income), the principal issues or problems of the country, the target population, and the main health disease or outcome of interest in the study.

To classify the type and frequency of indicators cited in the studies, the WHO monitoring framework proposed by the EQuAL project was used. Indicators used to assess negative impacts on health were classified and described, along with the main policy priorities and challenges associated with monitoring the SDH.

## Results

**Fig. 2:**
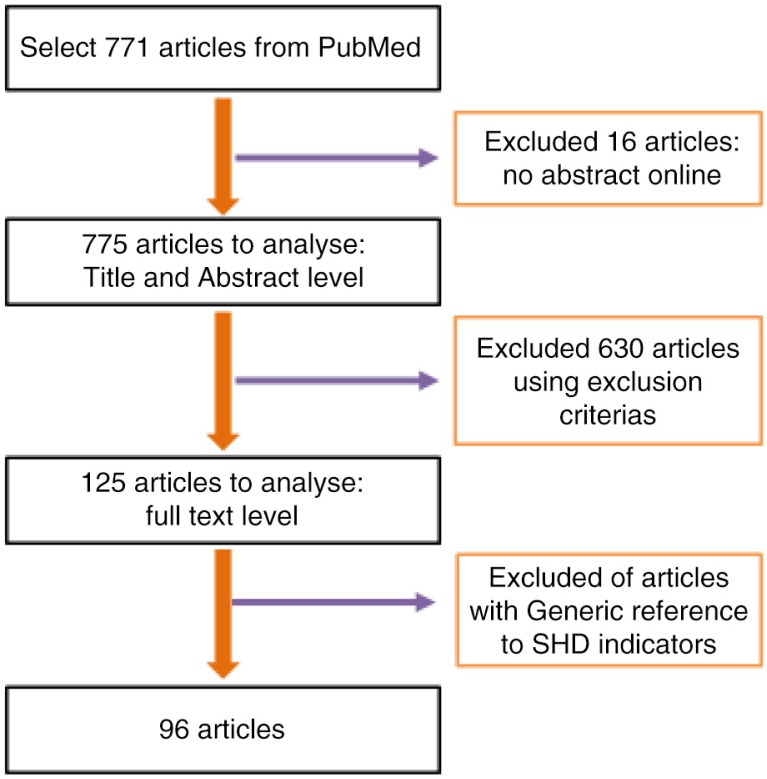
The scoping review selection process.

Our first search located 775 articles. After reviewing the titles and abstracts, 125 articles were selected. After the screening at full text level, 29 articles were excluded. The final sample consisted of 96 articles (Fig. 2) (Supplementary file).

### Profile of the international studies on monitoring SHD at the national/country level

**Graph 1:**
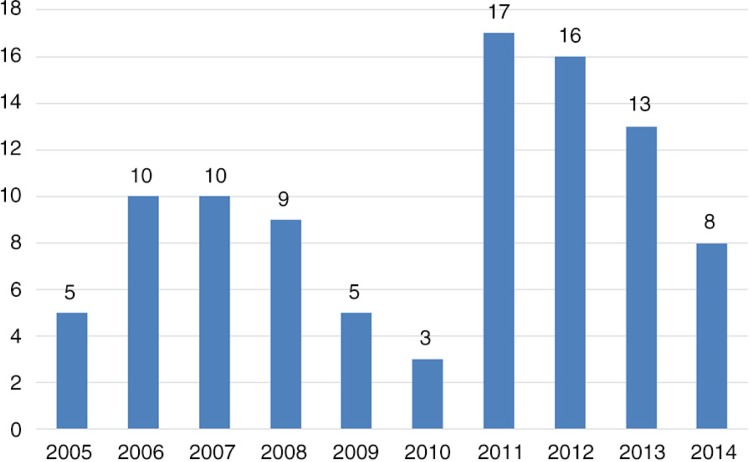
Scientific articles published by year.

Most of the articles were published in English (96%), during the preceding 5 years (59%), and after 2011, the year of the Rio Political Declaration (56%) (Graph 1). The majority of studies employed quantitative methods (93%). Most of the studies (73%) used multiple regression models to explain the relationship between social determinants and health conditions, some studies included geo-mapping techniques (6%), and 10% of studies used technical indexes, to describe socio-economic inequalities. The majority of the studies were cross-sectional in design (54%), and most of the visual displays used to illustrate the results were represented by a combination of tables (63%), graphics (19%), figures (11%), and maps (9%) (Table 1).

**Table 1:** Methodological approaches and visual displays

	*N*	%
Methodological approaches	96	
Mixed: quali–quanti	5	5
Qualitative	2	2
Quantitative	89	93
Statistical methods and techniques^a^	116	
Geo-mapping	6	6
Descriptive with bivariate regression	20	19
Mixed methods	2	2
Multivariate regression	76	73
Indexes	12	10
Cross-sectional studies	96	
Yes	52	54
No	44	46
Type of visual display^a^	142	
Figures	16	11
Graphs	27	19
Maps	13	9
Tables	84	59
No visual display	2	1

a The articles used one or more statistical methods or visual displays.

### The nation/country profile

**Graph 2:**
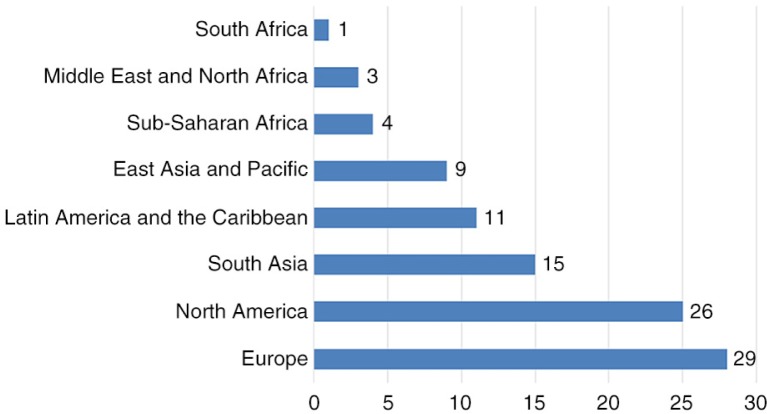
World regions of studies.

**Graph 3:**
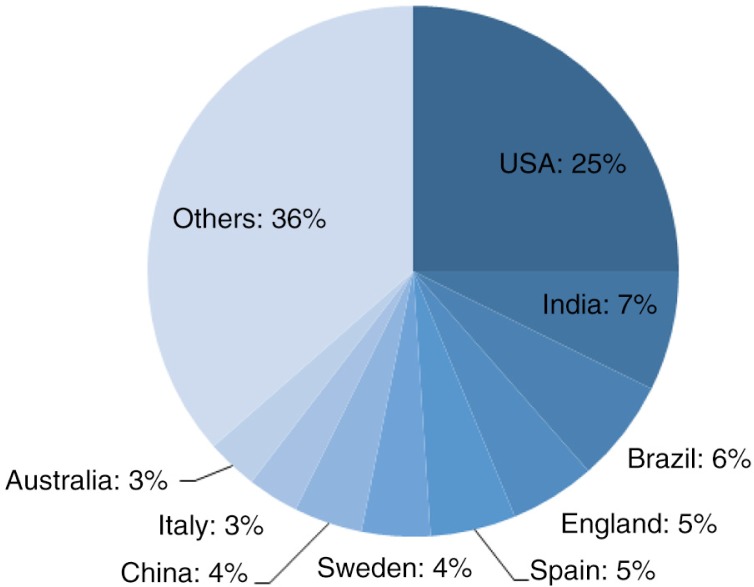
Countries of studies.

The majority of the research focused on Europe (29%), North America (26%), and Asia (24%) (Graph 2). Among the 38 nations analysed by published studies, the seven countries most frequently represented were the United States (25%), India (7%), Brazil (6%), England (5%), Spain (4%), Sweden (4%), and China (4%) (Graph 3).

A large proportion of the studies (65%) focused on high-income countries and a lower proportion (19%) on upper-middle income countries. In contrast, lower-middle income countries were included in 14% of the studies and low-income countries in just 2%. Studies outlined country contextual issues (n=169), which we grouped into five main categories: socio-demographic (35%), health (33%), economic (15%), and regional differences (15%) and political problems (2%) (Table 2). Assessment of these categories showed that in countries with socio-demographic problems, for example, the main issues were to do with sociocultural factors (73%), racial or ethnic discrimination (27%), social inequality (12%), socio-economic geographic differences (12%), and immigration (10%). In countries where health problems were taken into consideration, there was a larger focus on health system issues (44%) such as the lack of universal health care coverage (33%), limited access to healthcare services (33%) and health policy/program evaluation (10%), rather than on health population outcomes (24%). In countries with economic problems, the main focus was on high inequity and poverty within the population of the country (38%).

**Table 2:** Country context problems

	*N*	%
Social and demographic problems		
Race/ethnic inequality/discrimination	16	27
Socio-economic geographic differences	7	12
Cultural differences	3	5
Immigration	6	10
Social inequality/capital	7	12
Gender discrimination	4	7
Aging population	4	7
Life expectancy at birth	2	3
Population growth/density	2	3
Demographic characteristics	3	5
Others	5	8
Health issues		
Lack of (universal) healthcare/unequal access/utilisation	18	33
Health policy/program evaluation	6	11
Incidence/prevalence of specific disease	6	11
Chronic diseases and mortality	7	13
Child health	6	11
Maternal/women's health	5	9
Others	7	13
Economic issues		
Low economic condition of groups	5	21
Economic rapid grown	5	21
National-level economic problems	9	38
Regional inequality	5	21
Politic issues	4	2
Regional differences	26	15

When we evaluated the health disease/outcomes we observed that the vast majority of studies (69%) focused on specific health diseases (Table 3). Additionally, studies focused more on population groups (39%) than on the national population of the country as a whole (31%) (Table 3).

**Table 3:** Health disease/outcome and population target of the studies

	*N*	%
Type of health disease/outcome		
General health	31	31
Women's, maternal, and child health	20	20
Chronic disease and cancer	13	13
Mental health	12	12
Health system analysis	9	9
Oral health	8	8
External cause of injuries	3	3
Occupational health	3	3
Infectious diseases	1	1
Men's health	1	1
Population target		
General population	30	31
Children	17	17
Women	17	17
Adults	9	9
Adolescents	8	8
Multiracial/ethnic groups	6	6
Senior citizens	5	5
Workers	5	5
Sex minorities	1	1

### Domains and indicators of SDH and health inequity national monitoring

A total of 301 indicators were used in the 96 articles. Only a limited number of indicators corresponded exactly with those in the EQuAL domains (15%), but their distribution corresponded uniformly within all the 12 domains of the EQuAL framework (Table 4). Due to the fact that our interest was in the global representativeness of the indicators, the distribution of all indicators (EQuAL indicators and other indicators) cited in the studies (n=301) was analysed. This analysis resulted in a more polarised distribution compared to the *identical* indicators, which corresponded exactly to the EQuAL domains described in Table 4. Specifically, indicators were more representative of the *livelihoods and social protection* cluster domain (51%), rather than the domains of *environment quality* (15%) or *accountability and inclusion* (9%). The main reason for this difference was that the indicators were concentrated in 6 of the original 12 domains of the EQuAL framework (78%), *income and wealth* (28%) and *education and skills* (17%) being heavily represented primarily because of the frequent use of measures of income and education. A third class of indicators frequently used was *index/multiple measures for economic inequity/income* (10%) and another group was related to *race/ethnicity/immigrant status and conditions* (10%). This latter class of indicators was used more often than the EQuAL domain of *discrimination*. Domains such as *accountability and participation* and *intergenerational equity* were not considered in any of the studies (Table 4). Overall most studies focused on evaluating the impact of SDH on health population outcomes (70%), whereas a reduced number aimed at evaluating the impact of the SDH on health system services outcomes (20%) or evaluating health policy impacts on SDH (10%).

**Table 4:** Domains and indicators

	EQuAL indicators		Other indicators		Total		
			
	*n*	% relative	*n*	% relative	*n*	% relative	% total
A. Environment quality	14		31		45		15
A.1. Amenities	8	57	1	3	9	20	3
A.2. Community spaces and products	4	29	19	61	23	51	8
A.3. Housing	2	14	4	13	6	13	2
A.4. Working conditions	0	0	7	23	7	16	2
B. Accountability and inclusion	16		11		27		9
B.1. Accountability and participation	0	0	0	0	0	0	0
B.2. Discrimination	3	19	5	45	8	30	3
B.3. Gender equality	5	31	3	27	8	30	3
B.4. Social capital	8	50	3	27	11	41	4
C. Livelihoods and social protection	14		139		153		51
C.1. Education and skills	5	36	43	31	48	31	16
C.2. Employment relations	1	7	19	14	20	13	7
C.3. Income and wealth	8	57	77	55	85	56	28
C.4. Intergenerational equity	0	0	0	0	0	0	0
D. Other domains/indicators			76		76		25
D.1. Family structure/composition			13	17	13	17	4
D.2. Social cohesion/integration/protection			3	4	3	4	1
D.3. Index/multiple measures for economic inequity/income			29	38	29	38	10
D.4. Race/ethnicity/immigrant status			30	39	30	39	10
D.5. Healthcare utilisation			1	1	1	1	0
Total	44	15	257	85	301	100	

### The contribution of the indicators in the key mechanism of SDH or SDH pathways

As already noted, studies discussing causal models and pathways from social conditions to health inequities were frequent. The indicators that represented the main social determinants (main SDH) seen as having a negative impacting on health were classified into five main categories. The most common of these referred to living in a high discrimination context (35%), a place that involves social suffering, deprivation, and segregation for specific population groups based on race/ethnicity (53%), immigrant status (24%), and gender/sex (23%). This topic was mainly analysed at community levels. Second, living in financial hardship with low educational levels (31%) was commonly analysed in terms of high poverty/deprivation (71%) and low economic conditions (29%) and was generally considered at the individual/family level. Living in a low healthcare coverage level area (14%), living in urban–rural areas (14%), and work and employment conditions (6%) were less frequently covered in the studies.

### Set of social determinants indicators

Almost half of the published research (42%) described the relationship between social determinants and health through consideration of the main SDH pathways. These main pathways were typically described in association with a set of other social determinants represented by a high number of indicators (n=89) and were as follows.

Discrimination context was represented by a large number of indicators (n=51). The most frequent indicators were level of socio-economic status (59%), linguistic/cultural barriers to access to healthcare systems (47%), high racial/ethnic and gender segregation/discrimination of the area (41%), characteristics of rural deprived areas (24%), and economic barriers to access to healthcare system (24%).

Financial hardship/socio-economic level was represented by a lower number of indicators (n=26), mainly the following: the level of health coverage (43%), deprivation level of rural (36%) or urban (29%) area, family structure/composition (29%), and economic barriers to access to healthcare (21%).

The other three main social determinants were represented with fewer indicators (n=12) including health coverage level, linked to educational level and level of access to social protection programmes; rural–urban area differences, associated with rural deprivation levels and levels of industrial pollution of the environment; and work and employment conditions, associated with socio-economic status, occupational gender discrimination, and job/working conditions.

**Fig. 3:**
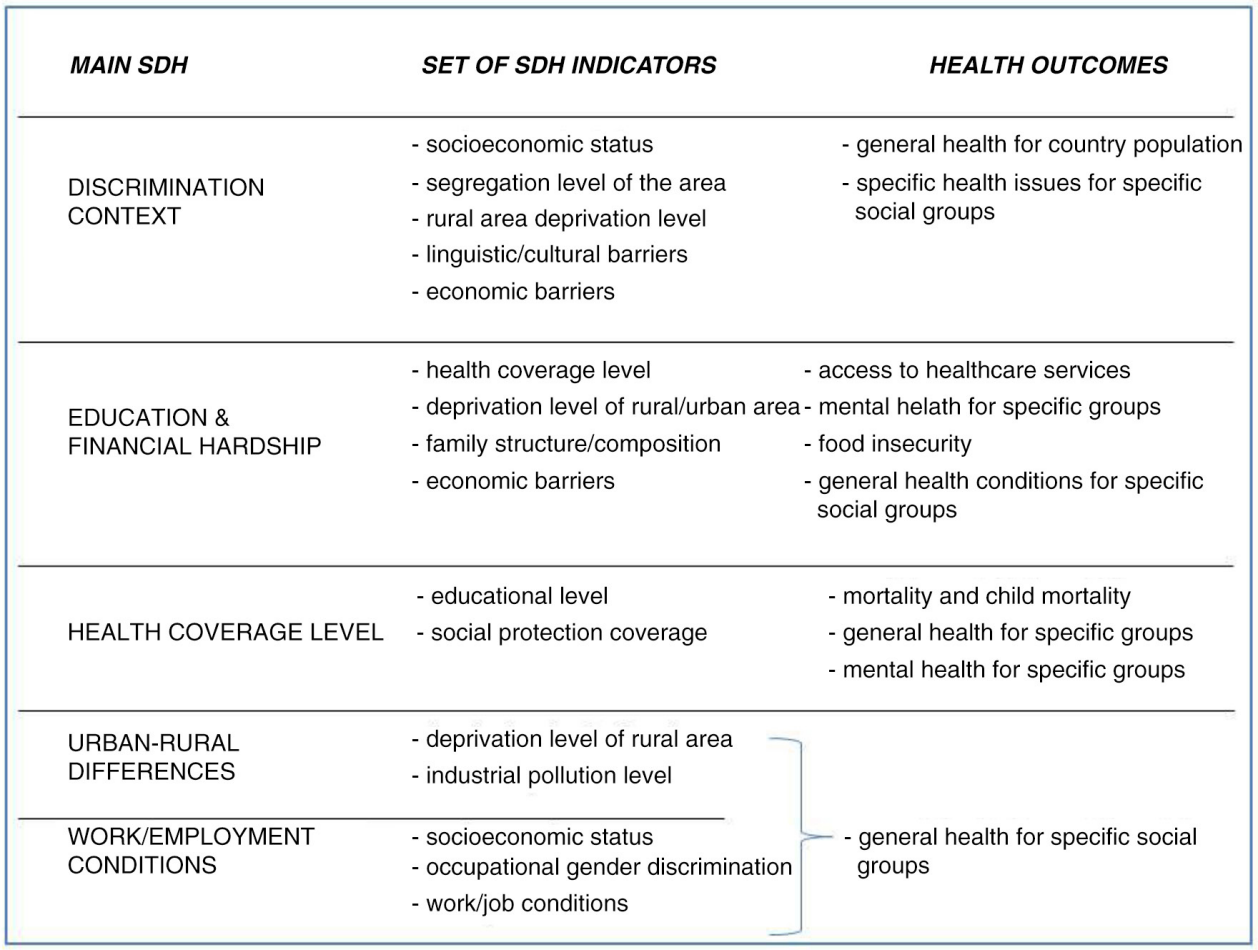
SDH pathways: main SDH, set of SDH indicators, and health outcomes.

In our study sample, more attention was given to general health conditions for specific groups (22%) and mental health for specific groups (19%) than to barriers to access to the health system (13%), general health conditions for the country population (10%), or mortality and child mortality (9%). For a summary of findings in the two last sections see Fig. 3, illustrating the main SDH pathways.

### Priorities and challenges of national SDH monitoring

*Policy priorities*: Several studies (n=65) highlighted policy priorities that were mainly directed at specific social groups. The most frequent themes were health promotion/literacy (25%), social promotion/protection (22%), and large socio-economic development interventions (20%) (Table 5).

**Table 5:** Policy priorities

	*N*	%
Social promotion/protection	14	22
Anti-discrimination	7	11
Health promotion/literacy	16	25
Intersectoral policies	10	15
Occupational policies	5	8
Large-scale socio-economic development interventions	13	20

*Priorities and needs of SDH monitoring*: In our evaluation, 61 items could benefit from more extensive measuring and monitoring within the EQuAL framework. These items were summarised into seven dimensions. Of these, the most commonly considered were socio-economic inequity (25%), population health conditions (21%), and health system conditions (16%). Subcategories are detailed in Table 6.

**Table 6:** Priorities and need for SDH monitoring

	*N*	%
Socio-economic inequity	15	
Specific population group conditions	6	40
Geographic differences	3	20
SDH impact at local level	3	20
Family/individual level	2	13
General population	1	7
Population health conditions	13	
Specific disease for low social condition groups	3	23
Ethnic/race/immigration group	3	23
Gender groups	2	15
Rural deprived groups	2	15
Local/family focus	2	15
Regional differences	1	8
Health system conditions	10	
Coverage associated with geographic characteristics (access)	4	40
Financial/ethnic/racial barriers to access	4	40
Quality of health services	1	10
Health promotion	1	10

*Key measures of SDH monitoring*: The studies also highlighted the importance of specific key indicators (n=24), mainly indexes and technical multilevel measures (82%) that were needed to represent mainly socio-economic inequity (54%) (Table 7).

**Table 7:** Key measures of SDH monitoring

Indicators of inequity	*n*	%	Multidimensional index
Socio-economic inequity	13	54	7
Gender inequity	4	17	2
Health inequity	2	8	0
Sociocultural inequity (local level)	4	17	2
Others	1	4	1
	24	100	12

## Discussion

This review showed that SDH and social inequity monitoring and evaluation using national indicators is well reported in the current scientific literature, independent of the economic level of the country and magnitude of deprivation within population groups. The importance of focusing on SDH national monitoring has been significantly influenced by the international political discussion on SDH, marked by the MDGs and the Rio Political Declaration of 2011.

A large number of the studies reviewed here used quantitative methods, including multilevel and cross-sectional analyses of SDH. Longitudinal studies are helpful in understanding the long-term impacts of the SDH on country populations and, given the importance of geographic location and health (e.g. urban–rural differences), geo-mapping techniques are useful. There is a paucity of published research using qualitative methods, and more studies of this type are needed.

We identified the frequent use of complex explanatory models considering multiple SDH dimensions that influence health population outcomes. We identified many studies that defined individual/family socio-economic status through multiple measures and indexes of socio-economic deprivation and financial hardship. This demonstrates a growing sophistication specifically in the construction of more complex multimeasures of socio-economic inequity, considered at different social aggregation levels, from the individual level to family, neighbourhood, community, municipal, and regional levels (8–10). There were also other indicators and indexes used to measure race and ethnic inequity (11–13) as well as gender inequity, especially in the context of work/employment (14) and family/social limitations (15, 16).

Independent of the development of new indicators or indexes (9, 17), most of the studies used traditional indicators, like income *per capita* and illiteracy rates. Further investigations are needed to confirm whether these traditional indicators are commonly available in secondary data. Given the wide range of social dimensions that impact on health, issues of coverage in primary data sets also need exploration.

In studies relating to health outcomes and to the population target of the studies there was a focus on measuring prevalence for specific health diseases in population groups. These studies focused more on the social conditions of marginalised or discriminated groups rather than on individual social conditions. Monitoring a broad range of social indicators is considered a central issue in development, as demonstrated by the creation of two specific sustainable development goals (SDGs) (17.18 and 17.19) that refer specifically to data monitoring and accountability in the 2030 Agenda for Sustainable Development (18). Moreover, monitoring the health conditions of a population is an essential process of every national healthcare system: not only does it allow the understanding of whether population health is improving or worsening, but it could also indicate the effectiveness of public interventions (19). Particularly in low- and middle-income countries, such monitoring can reveal reductions or increases in health inequalities and the impact of social policies. The process of monitoring health outcomes and socio-economic health inequalities, by using stand-alone indicators or stratifiers and adjusting variables for health outcomes, is becoming more important in public health. Monitoring frameworks can be useful to identify and classify indicators according to a predefined rationale (20). The EQuAL framework assembled measures and indicators of health determinants and of the barriers they can cause in terms of access to healthcare services. The domains and indicators selected were not only technically feasible but were able to collect reliable measures, as shown in this review. Importantly they are also easy to understand and relevant for policy-makers (6).

This could be one reason why some indicators were not easily found in the academic literature covered in this review. It would be useful to undertake a content analysis of these indicators from a policy perspective. This could inform the framework for national monitoring of SDH (21).

Our review showed that some dimensions and domains of SDH were given less attention than others, while some were not included. There is a need for improved interdisciplinary practices in order to define multiple measures, for more comprehensive, comparable, and standardised SDH monitoring research. Interdisciplinary research is important. The explanation of natural and social phenomena requires broad understanding across many different social and scientific fields (22, 23). This observation is relevant to public health-related studies as well as for monitoring operational practices, especially when dealing with the SDH and intersectoral policy-making processes (24).

One of the main limitations of our review was the use of a single database. The database used was the broadest and most relevant for the biomedical literature. However we acknowledge that the review would potentially have been more informative with the inclusion of other relevant databases. Second, our analysis was limited to scientific articles and excluded the grey literature. It also prioritised peer-reviewed studies with higher quality standards and did not include policy and institutional reports. Third, the prevalence of high-income countries in our country profile sample, particularly the United States, influenced some of our results, for example the characterisation of the main national context problems as sociocultural issues, rather than as strictly economic issues. Studies on national health problems were limited by available data and resources on health and healthcare for specific social groups.

## Conclusions

Our review illustrated that the attention on SDH monitoring has grown in terms of its importance and complexity within the scientific health literature. We highlighted the importance of the move towards a more holistic, interdisciplinary, and intersectoral perspective for scientific research as well as for policy.

The set of indicators proposed in the EQuAL project is the result of a health exploration research model that uses qualitative and quantitative methodologies to expand the range of possible measures and indicators for monitoring the SDH. This approach is important because it helps to identify factors that influence the rights of all citizens to achieve good health. Evidence from this process can be used to inform equity-focused public policies.

## Supplementary Material

Scoping review: national monitoring frameworks for social determinants of health and health equity
