# Supplementary material for: Scoping review: national monitoring frameworks for social determinants of health and health equity
Source: Glob Health Action. 2016 Feb 5;9:10.3402/gha.v9.28831. doi: 10.3402/gha.v9.28831 (PMC4744868; doi:10.3402/gha.v9.28831)
Supplement: Scoping review: national monitoring frameworks for social determinants of health and health equity [file GHA-9-28831-s001.docx]

**the scoping review articles**

1. Acosta-Ramirez N, Rodriguez-Garcia J. Inequity in infant vaccination coverage in Colombia 2000 and 2003. Rev Salud Publica (Bogota) 2006; 8(1):102-15.
2. Adams AM, Rabbani A, Ahmed S, Mahmood SS, Al-Sabir A, Rashid SF, et al. Explaining equity gains in child survival in Bangladesh: scale, speed, and selectivity in health and development. Lancet 2013;382(9909):2027-37.
3. Agarwal S, Srivastava A. Social determinants of children's health in urban areas in India. J Health Care Poor Underserved 2009;20(4):68-89.
4. Ahnquist J, Fredlund P, Wamala SP. Is cumulative exposure to economic hardships more hazardous to women's health than men's? A 16-year follow-up study of the Swedish Survey of Living Conditions. J Epidemiol Community Health 2007;61(4):331-6.
5. Ahnquist J, Wamala SP, Lindstrom M. Social determinants of health--a question of social or economic capital? Interaction effects of socioeconomic factors on health outcomes. SocSci Med 2012;74(6):930-9.
6. Antunes JL, Peres MA, de Campos Mello TR, Waldman EA. Multilevel assessment of determinants of dental caries experience in Brazil. Community Dent Oral Epidemiol 2006; 34(2):146-52.
7. Areai DM, Thomson WM, Foster Page LA, Denny SJ, Crengle S, Clark TC, al. Self-reported oral health, dental self-care and dental service use among New Zealand secondary school students: findings from the Youth 07 study. N Z Dent J 2011;107(4):121-6.
8. Atherton K, Power C. Health inequalities with the National Statistics-Socioeconomic classification: disease risk factors and health in the 1958 British birth cohort. Eur J Public Health 2007; 17(5):486-91.
9. Bang JY, Yadegarfar G, Soljak M, Majeed A. Primary care factors associated with cervical screening coverage in England. J Public Health (Oxf) 2012; 34(4):532-8.
10. Bedrouni M. Regionalisation of the Millennium Development Goals: markers for assessing child health in Algeria. Cahiers Santé 2009;19(3):149-57.
11. Beltran VM, Harrison KM, Hall HI, Dean HD. Collection of social determinant of health measures in U.S. national surveillance systems for HIV, viral hepatitis, STDs, and TB. Public Health Rep 2011; 126(3):41-53.
12. Benyamini Y, Boyko V, Blumstein T, Lerner-Geva L. Health, cultural and socioeconomic factors related to self-rated health of long-term Jewish residents, immigrants, and Arab women in midlife in Israel. Women Health 2014; 54(5):402-24.
13. Bhutta ZA, Hafeez A, Rizvi A, Ali N, Khan A, Ahmad F, et al. Reproductive, maternal, newborn, and child health in Pakistan: challenges and opportunities. Lancet 2013; 381(9884):2207-18.
14. Biswas S, Bose K. Effect of number of rooms and sibs on nutritional status among rural Bengalee preschool children from eastern India. CollAntropol 2011;35(4):1017-22.
15. Bockerman P, Johansson E, Helakorpi S, Uutela A. Economic inequality and population health: looking beyond aggregate indicators. Sociol Health Illn 2009;31(3):422-40.
16. Bronnum-Hansen H, Baadsgaard M. Increase in social inequality in health expectancy in Denmark. Scand J Public Health 2008;36(1):44-51.
17. Campos-Serna J, Ronda-Perez E, Artazcoz L, Benavides FG. Gender inequalities in occupational health in Spain. GacSanit 2012;26(4):343-51.
18. Carlson P. Relatively poor, absolutely ill? A study of regional income inequality in Russia and its possible health consequences. J Epidemiol Community Health 2005;59(5):389-94.
19. Castor ML, Smyser MS, Taualii MM, Park AN, Lawson SA, Forquera RA. A nationwide population-based study identifying health disparities between American Indians/Alaska Natives and the general populations living in select urban counties. Am J Public Health 2006;96(8):1478-84.
20. Cavalini LT, de Leon AC. Morbidity and mortality in Brazilian municipalities: a multilevel study of the association between socioeconomic and healthcare indicators. Int J Epidemiol 2008;37(4):775-83.
21. Chen J, Chen S, Landry PF. Migration, environmental hazards, and health outcomes in China. SocSci Med 2013;80:85-95.
22. Chen J, Vargas-Bustamante A. Estimating the effects of immigration status on mental health care utilizations in the United States. J Immigr Minor Health 2011;13(4):671-80.
23. Chen X, Gelaye B, Williams MA. Sleep characteristics and health-related quality of life among a national sample of American young adults: assessment of possible health disparities. Qual Life Res 2014;23(2):613-25.
24. Cheng NF, Han PZ, Gansky SA. Methods and software for estimating health disparities: the case of children's oral health. Am J Epidemiol 2008;168(8):906-14.
25. Cherutich P, Kaiser R, Galbraith J, Williamson J, Shiraishi RW, Ngare C, et al. Lack of knowledge of HIV status a major barrier to HIV prevention, care and treatment efforts in Kenya: results from a nationally representative study. PLoS One 2012;7(5):e36797.
26. Choi Y, Bishai D, Hill K. Socioeconomic differentials in supplementation of vitamin A: evidence from the Philippines. J Health PopulNutr 2005; 23(2):156-64.
27. Clark RA, Driscoll A, Nottage J, McLennan S, Coombe DM, Bamford EJ, et al. Inequitable provision of optimal services for patients with chronic heart failure: a national geo-mapping study. Med J Aust 2007;186(4):169-73.
28. da Cunha SS, Pujades-Rodriguez M, Barreto ML, Genser B, Rodrigues LC. Ecological study of socioeconomic indicators and prevalence of asthma in schoolchildren in urban Brazil.BMC Public Health 2007;7:205.
29. De Vogli R, Mistry R, Gnesotto R, Cornia GA. Has the relation between income inequality and life expectancy disappeared? Evidence from Italy and top industrialised countries. J Epidemiol Community Health 2005;59(2):158-62.
30. Denboba D, McPherson MG, Kenney MK, Strickland B, Newacheck PW. Achieving family and provider partnerships for children with special health care needs. Pediatrics 2006;118(4):1607-15.
31. Dey AN, Lucas JW. Physical and mental health characteristics of U.S.- and foreign-born adults: United States, 1998-2003. Adv Data 2006(369):1-19.
32. Ettarh RR, Kyobutungi C. Physical access to health facilities and contraceptive use in Kenya: evidence from the 2008-2009 Kenya Demographic and Health Survey. Afr J Reprod Health 2012;16(3):48-56.
33. Fang P, Dong S, Xiao J, Liu C, Feng X, Wang Y. Regional inequality in health and its determinants: evidence from China. Health Policy 2010;94(1):14-25.
34. Fekete C, Siegrist J, Reinhardt JD, Brinkhof MW. Is financial hardship associated with reduced health in disability? The case of spinal cord injury in Switzerland. PLoS One 2014;9(2):e90130.
35. Flores G, Lin H. Trends in racial/ethnic disparities in medical and oral health, access to care, and use of services in US children: has anything changed over the years? Int J Equity Health 2013;12:10.
36. Gething PW, Johnson FA, Frempong-Ainguah F, Nyarko P, Baschieri A, Aboagye P, et al. Geographical access to care at birth in Ghana: a barrier to safe motherhood. BMC Public Health 2012;12:991.
37. Gilbert CE, Shah SP, Jadoon MZ, Bourne R, Dineen B, Khan MA, et al. Poverty and blindness in Pakistan: results from the Pakistan national blindness and visual impairment survey. BMJ 2008;336(7634):29-32.
38. Gong F, Xu J, Takeuchi DT. Beyond conventional socioeconomic status: examining subjective and objective social status with self-reported health among Asian immigrants. J Behav Med 2012;35(4):407-19.
39. Grann V, Troxel AB, Zojwalla N, Hershman D, Glied SA, Jacobson JS. Regional and racial disparities in breast cancer-specific mortality. SocSci Med 2006;62(2):337-47.
40. Guiotoku SK, Moyses ST, Moyses SJ, Franca BH, Bisinelli JC. Racial inequity in oral health in Brazil. Rev PanamSaludPublica 2012;31(2):135-41.
41. Habicht J, Xu K, Couffinhal A, Kutzin J. Detecting changes in financial protection: creating evidence for policy in Estonia. Health Policy Plan 2006;21(6):421-31
42. Haider AH, Hashmi ZG, Zafar SN, Hui X, Schneider EB, Efron DT, et al. Minority trauma patients tend to cluster at trauma centers with worse-than-expected mortality: can this phenomenon help explain racial disparities in trauma outcomes? Ann Surg 2013;258(4):572-9; discussion 9-81.
43. Harris KM, Gordon-Larsen P, Chantala K, Udry JR. Longitudinal trends in race/ethnic disparities in leading health indicators from adolescence to young adulthood. Arch PediatrAdolesc Med 2006;160(1):74-81.
44. Hatzenbuehler ML, Bellatorre A, Lee Y, Finch BK, Muennig P, Fiscella K. Structural stigma and all-cause mortality in sexual minority populations. SocSci Med 2014;103:33-41.
45. Hawthorne G, Korn S, Richardson J. Population norms for the AQoL derived from the 2007 Australian National Survey of Mental Health and Wellbeing. Aust N Z J Public Health 2013;37(1):7-16.
46. Huang KY, Calzada E, Cheng S, Brotman LM. Physical and mental health disparities among young children of Asian immigrants. J Pediatr 2012;160(2):331-6.e1.
47. Huang N, Yip W, Chang HJ, Chou YJ. Trends in rural and urban differentials in incidence rates for ruptured appendicitis under the National Health Insurance in Taiwan. Public Health 2006;120(11):1055-63.
48. Hurtado D, Kawachi I, Sudarsky J. Social capital and self-rated health in Colombia: the good, the bad and the ugly. SocSci Med 2011;72(4):584-90.
49. Hystad P, Carpiano RM, Demers PA, Johnson KC, Brauer M. Neighbourhood socioeconomic status and individual lung cancer risk: evaluating long-term exposure measures and mediating mechanisms. SocSci Med. 2013;97:95-103.
50. Joe W, Mishra US, Navaneetham K. Socio-economic inequalities in child health: recent evidence from India. Glob Public Health 2010;5(5):493-508.
51. Johnson-Lawrence V, Griffith DM, Watkins DC. The effects of race, ethnicity, and mood/anxiety disorders on the chronic physical health conditions of men from a national sample. Am J Mens Health 2013;7(4):58-67.
52. Kim IH, Khang YH, Cho SI, Chun H, Muntaner C. Gender, professional and non-professional work, and the changing pattern of employment-related inequality in poor self-rated health, 1995-2006 in South Korea. J Prev Med Public Health 2011; 44(1):22-31.
53. Kim JK, Baker LA, Seirawan H, Crimmins EM. Prevalence of oral health problems in U.S. adults, NHANES 1999-2004: exploring differences by age, education, and race/ethnicity. Spec Care Dentist 2012; 32(6):234-41.
54. Kivits J, Erpelding ML, Guillemin F. Social determinants of health-related quality of life. Rev EpidemiolSantePublique 2013; 61 Suppl 3:S189-94.
55. Klanšček HJ, Ziberna J, Korošec A, Zurc J, Albreht T. Mental health inequalities in Slovenian 15-year-old adolescents explained by personal social position and family socioeconomic status. Int J Equity Health. 2014 Mar 28;13:26.
56. Kramer MR, Hogue CR. Place matters: variation in the black/white very preterm birth rate across U.S. metropolitan areas, 2002-2004. Public Health Rep 2008;123(5):576-85.
57. Laflamme L, Vaez M. Car crash and injury among young drivers: contribution of social, circumstantial and car attributes. Int J InjContrSafPromot 2007;14(1):5-10.
58. Lau M, Lin H, Flores G. Racial/ethnic disparities in health and health care among U.S. adolescents. Health Serv Res 2012;47(5):2031-59.
59. Li X, Zhu J, Wang Y, Mu D, Dai L, Zhou G, et al. Geographic and urban-rural disparities in the total prevalence of neural tube defects and their subtypes during 2006-2008 in China: a study using the hospital-based birth defects surveillance system. BMC Public Health 2013;13:161.
60. Liang Y, Lu P. Medical insurance policy organized by Chinese government and the health inequity of the elderly: longitudinal comparison based on effect of New Cooperative Medical Scheme on health of rural elderly in 22 provinces and cities. Int J Equity Health 2014;13:37.
61. Lima-Costa MF, Matos DL. Prevalence and factors associated with mammograms in the 50-69-year age group: a study based on the Brazilian National Household Sample Survey (PNAD-2003). Cad SaudePublica 2007;23(7):1665-73.
62. Mansdotter A, Lindholm L, Lundberg M, Winkvist A, Ohman A. Parental share in public and domestic spheres: a population study on gender equality, death, and sickness. J Epidemiol Community Health 2006; 60(7):616-20.
63. Mashal T, Nakamura K, Kizuki M, Seino K, Takano T. Impact of conflict on infant immunisation coverage in Afghanistan: a countrywide study 2000-2003. Int J Health Geogr 2007;6:23.
64. McLaughlin KA, Green JG, Alegria M, Jane Costello E, Gruber MJ, Sampson NA, et al. Food insecurity and mental disorders in a national sample of U.S. adolescents. J Am Acad Child Adolesc Psychiatry 2012;51(12):1293-303.
65. McLaughlin KA, Xuan Z, Subramanian SV, Koenen KC. State-level women's status and psychiatric disorders among US women. Soc Psychiatry PsychiatrEpidemiol 2011;46(11):1161-71.
66. Mejia G, Armfield JM, Jamieson LM. Self-rated oral health and oral health-related factors: the role of social inequality. Aust Dent J. 2014;59(2):226-33.
67. Minardi V, Campostrini S, Carrozzi G, Minelli G, Salmaso S. Social determinants effects from the Italian risk factor surveillance system PASSI. Int J Public Health 2011;56(4):359-66.
68. Mohanty SK. Multidimensional poverty and child survival in India. PLoS One 2011;6(10):e26857.
69. Mohnen SM, Groenewegen PP, VÃ¶lker B, Flap H. Neighborhood social capital and individual health. Soc Sci Med. 2011 Mar;72(5):660-7.
70. Mohnen SM, Groenewegen PP, Volker B, Flap H. Neighbourhood social capital and individual health. SocSci Med 2011;72(5):660-7.
71. Morello-Frosch R, Jesdale BM. Separate and unequal: residential segregation and estimated cancer risks associated with ambient air toxics in U.S. metropolitan areas. Environ Health Perspect 2006;114(3):386-93.
72. Niedhammer I, Chastang JF, David S, Kelleher C. The contribution of occupational factors to social inequalities in health: findings from the national French SUMER survey. SocSci Med 2008;67(11):1870-81.
73. Nikiema B, Haddad S, Potvin L. Measuring women's perceived ability to overcome barriers to healthcare seeking in Burkina Faso. BMC Public Health 2012;12:147.
74. Oka K, Shibata A. Determinants of meeting the public health recommendations for physical activity among community-dwelling elderly Japanese. Curr Aging Sci 2012;5(1):58-65.
75. Perez-Cueto FJ, Naska A, Monterrey J, Almanza-Lopez M, Trichopoulou A, Kolsteren P. Monitoring food and nutrient availability in a nationally representative sample of Bolivian households. Br J Nutr 2006;95(3):555-67.
76. Perez-Rodrigo C, Aranceta Bartrina J, Serra Majem L, Moreno B, Delgado Rubio A. Epidemiology of obesity in Spain. Dietary guidelines and strategies for prevention. Int J VitamNutr Res 2006;76(4):163-71.
77. Prasad AM, Chakraborty G, Yadav SS, Bhatia S. Addressing the social determinants of health through health system strengthening and inter-sectoral convergence: the case of the Indian National Rural Health Mission. Glob Health Action 2013;6:1-11.
78. Riva M, Curtis S, Gauvin L, Fagg J. Unravelling the extent of inequalities in health across urban and rural areas: evidence from a national sample in England. SocSci Med 2009;686(4):654-63.
79. Rocha KB, Muntaner C, Gonzalez Rodriguez MJ, Baksai PB, Vallebuona C, Borrell C, et al. Sial class, health inequalities, and health-related behaviors of working people in Chile. Rev Panam Salud Publica 2013;33(5):340-8.
80. Rosales-Lopez A, Ortiz-Posadas MR. An indicator to estimate the access to imaging services in the Costa Rican public health system. J Digit Imaging 2014;27(1):41-8.
81. Ruiz-Munoz D, Perez G, Garcia-Subirats I, Diez E. Social and economic inequalities in the use of contraception among women in Spain. J Womens Health (Larchmt) 2011;20(3):403-11.
82. Ruiz-Munoz D, Wellings K, Castellanos-Torres E, Alvarez-Dardet C, Casals-Cases M, Perez G. Sexual health and socioeconomic-related factors in Spain. Ann Epidemiol 2013;23(10):620-8.
83. Sabbah W, Watt RG, Sheiham A, Tsakos G. The role of cognitive ability in socio-economic inequalities in oral health. J Dent Res 2009;88(4):351-5.
84. Scarselli A, Di Marzio D, Marinaccio A, Iavicoli S. The register of exposed workers to carcinogens: legislative framework and data analysis. Med Lav 2010;101(1):9-18.
85. Silver D, Mijanovich T, Uyei J, Kapadia F, Weitzman BC. Lifting boats without closing gaps: child health outcomes in distressed US cities from 1992-2002. Am J Public Health 2011;101(2):278-84.
86. Smith ME, Tawiah EO, Badasu DM. Why some women deliver in health institutions and others do not: a cross sectional study of married women in Ghana, 2008. Afr J Reprod Health. 2012;16(3):36-47.
87. Somkotra T, Detsomboonrat P. Is there equity in oral healthcare utilization: experience after achieving Universal Coverage. Community Dent Oral Epidemiol 2009;37(1):85-96.
88. Timmermans S, Bonsel GJ, Steegers-Theunissen RP, Mackenbach JP, Steyerberg EW, Raat H, et al. Individual accumulation of heterogeneous risks explains perinatal inequalities within deprived neighbourhoods. Eur J Epidemiol 2011;26(2):165-80.
89. Tong SF, Ho C, Tan HM. Managing the aging man in Asia: A review. International Journal of Urology 2011;18(1):32-42.
90. Tsakos G, Demakakos P, Breeze E, Watt RG. Social gradients in oral health in older adults: findings from the English longitudinal survey of aging. Am J Public Health 2011;101(10):1892-9.
91. Vives-Cases C, Ruiz-Cantero MT, Escriba-Aguir V, Miralles JJ. The effect of intimate partner violence and other forms of violence against women on health. J Public Health (Oxf) 2011;33(1):15-21.
92. Volkers AC, Westert GP, Schellevis FG. Health disparities by occupation, modified by education: a cross-sectional population study. BMC Public Health 2007;7:196.
93. Westphal MF, Zioni F, Almeida MF, Nascimento PR. Monitoring Millennium Development Goals in Brazilian municipalities: challenges to be met in facing up to iniquities. Cad SaudePublica 2011;27(2):S155-63.
94. Williams DR, Gonzalez HM, Williams S, Mohammed SA, Moomal H, Stein DJ. Perceived discrimination, race and health in South Africa. SocSci Med 2008; 67(3):441-52.
95. Zhao G, Ford ES, Tsai J, Li C, Ahluwalia IB, Pearson WS, et al. Trends in health-related behavioral risk factors among pregnant women in the United States: 2001-2009. J Womens Health (Larchmt) 2012; 21(3):255-63.
96. Zimmer Z, Prachuabmoh V. Comparing the socioeconomic status--health gradient among adults 50 and older across rural and urban areas of Thailand in 1994 and 2007. Soc Sci Med 2012;74(12):1921-8.
